# Dilution‐Driven Regulation of Biostimulatory Signaling in Calcium Hydroxylapatite–PRGF Systems: Implications for Early Dermal Remodeling

**DOI:** 10.1111/jocd.71257

**Published:** 2026-10-25

**Authors:** Eduardo Anitua, Roberto Tierno, Mohammad Hamdan Alkhraisat

**Affiliations:** ^1^ BTI Biotechnology Institute Vitoria Spain; ^2^ University Institute for Regenerative Medicine & Oral Implantology, UIRMI (UPV/EHU‐Fundación Eduardo Anitua) Vitoria Spain

**Keywords:** calcium hydroxyapatite, dermal fillers, extracellular matrix remodeling, plasma rich in growth factors (PRGF), skin regeneration

## Abstract

**Background:**

Calcium hydroxylapatite (CaHA)‐based fillers act as both volumizing agents and bioactive scaffolds capable of inducing matrix synthesis and dermal remodeling. Their rheological properties and biological activity can be modulated via dilution strategies, expanding their applicability in regenerative and aesthetic medicine. Plasma rich in growth factors (PRGF), a leukocyte‐poor autologous platelet concentrate, represents a biologically active diluent that may modulate the biological response to CaHA. This study evaluated key In vitro biological effects of diluting Radiesse, a biostimulatory soft‐tissue filler composed primarily of synthetic CaHA microspheres suspended in an aqueous carboxymethylcellulose gel carrier, with different PRGF formulations at clinically relevant ratios.

**Methods:**

Adhesion and proliferation assays, together with gene expression analyses of key extracellular matrix (ECM) remodeling markers, were conducted in human dermal fibroblasts (HDFs) in order to evaluate the biological response of primary human dermal fibroblasts to undiluted and PRGF‐diluted Radiesse formulations at 24 and 72 h.

**Results:**

The incorporation of PRGF significantly modulated the biological response of HDFs when compared with undiluted CaHA in a dilution‐ and time‐dependent manner. Relative to undiluted CaHA, PRGF‐based conditions enhanced fibroblast adhesion and proliferation. Gene expression analyses revealed a temporally regulated extracellular matrix (ECM) remodeling profile, characterized by a reduction in collagen expression, an increase in FGF2 transcripts at 72 h and an earlier increase in MMP1 mRNA. CTGF transcript levels remained largely unchanged following dilution, suggesting attenuation of sustained profibrotic signaling. However, functional outcomes, including cell adhesion and proliferation, exceeded the magnitude predicted by transcriptional changes.

**Conclusions:**

PRGF dilution modulates the early biological activity of CaHA‐based fillers by promoting fibroblast adhesion and proliferation while shifting extracellular matrix remodeling toward a dynamic turnover state, supporting its potential as a biologically active strategy to optimize regenerative endpoints. These findings support further investigation of PRGF as a biologically active diluent for CaHA‐based fillers.

## 1 Introduction

Calcium hydroxylapatite (CaHA)‐based biomaterials have been widely used in aesthetic and regenerative medicine due to their dual functionality as volumizing fillers and biostimulatory scaffolds [1]. Radiesse is a clinically established injectable dermal filler composed of CaHA microspheres (approximately 25–45 μm) suspended in an aqueous carboxymethylcellulose gel carrier, which provides immediate volumetric correction followed by long‐term biostimulatory effects after gel resorption [2, 3]. Beyond their immediate volumizing effect, CaHA microspheres have been shown to actively modulate dermal fibroblast behavior, promoting extracellular matrix (ECM) synthesis and long‐term tissue remodeling [4]. Histological and In vivo studies have consistently demonstrated that CaHA induces neocollagenesis, tissue remodeling, and dermal thickening, supporting its classification as a bioactive scaffold rather than a passive filler [5, 6, 7]. These effects are mediated by combined mechanical cues and cell–material interactions that activate fibroblast mechanotransduction pathways, leading to downstream signaling associated with extracellular matrix remodeling and transient TGF‐*β*–related profibrogenic responses [8].

Radiesse is commonly diluted to modulate its rheological and biological behavior, allowing a controlled transition from a primarily volumizing implant to a biostimulatory injectable tailored for superficial tissue remodeling [9, 10, 11, 12]. Dilution decreases product viscosity and microsphere concentration per injection volume, improving injectability, spreadability, and homogeneity within the dermal and subdermal planes while increasing the relative surface area of CaHA particles available for cell–material interaction [13, 14, 15]. As a result, diluted or hyperdiluted formulations reduce focal volume expansion but enhance fibroblast exposure to CaHA, favoring extracellular matrix remodeling and neocollagenesis rather than structural augmentation [16]. Clinically, Radiesse is typically diluted with sterile 0.9% sodium chloride solution, often combined with lidocaine for analgesia [10, 13, 14, 15, 17, 18, 19], whereas in emerging regenerative approaches it may also be mixed with autologous carriers such as platelet‐rich plasma (PRP), which can further influence cellular signaling and tissue repair responses [4, 20].

Plasma rich in growth factors (PRGF) is a leukocyte‐poor, standardized subtype of PRP, obtained via autologous blood centrifugation and characterized by a moderate increase in platelet concentration with negligible leukocyte content [21]. This formulation is designed to optimize the regenerative potential of bioactive molecules while minimizing the pro‐inflammatory and proteolytic activity typically associated with leukocyte‐rich preparations [22, 23]. PRGF is characterized by the presence of platelet‐derived bioactive mediators, including platelet‐derived growth factor (PDGF), transforming growth factor‐*β* (TGF‐*β*), vascular endothelial growth factor (VEGF), epidermal growth factor (EGF), hepatocyte growth factor (HGF), or insulin‐like growth factor (IGF), which collectively regulate cell proliferation, migration, angiogenesis, and ECM remodeling [24]. However, the biological activity of PRGF is critically dependent on its manufacturing protocol, activation state, and fibrin architecture, which determine the release kinetics of soluble factors and cell–matrix interactions. As a result, different PRGF‐derived formulations exhibit significant variability regarding mechanical and biological properties, which translate into distinct cellular responses and clinically relevant differences in tissue regeneration outcomes, thus rendering PRGF A highly versatile platform capable of addressing a wide range of clinical requirements [25, 26]. In this sense, scientific evidence derived from the biomedical literature supports its use across multiple dermatological and aesthetic conditions, including skin rejuvenation, wound healing, alopecia, scar management, and tissue regeneration [27, 28, 29, 30].

From a mechanistic perspective, the combination of structural biomaterials with autologous biological formulations represents a promising strategy to modulate regenerative outcomes [31]. In this context, the integration of CaHA‐based scaffolds with PRGF may provide a dual mode of action in which the material‐driven mechanotransductive activation of fibroblasts is complemented by the biochemical signaling mediated by platelet‐derived effectors [20]. Previous studies have demonstrated that the effectors contained in PRGF can modulate fibroblast phenotype, promoting a pro‐regenerative state characterized by balanced ECM deposition, controlled proteolysis, and reduced fibrotic signaling [32, 33, 34, 35]. However, despite the increasing clinical use of diluted and hyperdiluted CaHA formulations, particularly in combination with biological adjuvants, the mechanistic interplay between scaffold and diluent properties, dilution strategies, and biological responses remains incompletely understood. In particular, the extent to which different PRGF formulations modulate the rheological behavior of CaHA‐based formulations and reprogram fibroblast transcriptional activity in a dose‐ and time‐dependent manner has not been systematically investigated.

In this context, the present study aims to provide a comprehensive characterization of the in vitro biological effects of Radiesse diluted in PRGF across clinically relevant dilution ratios. By integrating functional assays and gene expression profiling in primary human dermal fibroblasts (HDFs), this work aims to elucidate the mechanistic basis underlying the interaction between CaHA‐based biomaterials and PRGF‐derived soluble effectors in order to evaluate the suitability of different PRGF formulations as biostimulatory diluents for Radiesse and to determine the optimal dilution ratios according to the specific clinical application, the intended mode of action, and the targeted biological effect. Specifically, we hypothesize that PRGF enhances the biostimulatory activity of Radiesse, promoting a rapid transition toward a pro‐regenerative cellular phenotype through the coordinated regulation of cell proliferation, active ECM remodeling, and growth factor signaling.

## 2 Materials and Methods

### 2.1 PRGF Obtention

After obtaining informed consent, blood samples were collected from three healthy volunteers into 9 mL tubes containing 0.4 mL of 3.8% (w/v) trisodium citrate as an anticoagulant. The samples were centrifuged at room temperature using a BTI System V centrifuge (BTI, Vitoria, Spain), and the PRGF fraction was carefully isolated while avoiding contamination from the leukocyte‐rich buffy coat. Cellular parameters of whole blood and PRGF were determined using a PENTRA ES60 hematology analyzer (HORIBA, Montpellier, France). Then, PRGF was divided into two equal fractions. One fraction was activated as described by Anitua et al. [26] and immediately combined with Radiesse according to the selected dilution strategy as detailed in the following section, whereas the other was activated and incubated for 1 h to enable coagulation, fibrin network formation, and subsequent release of the PRGF Supernatant prior to diluting Radiesse with the released fraction.

### 2.2 Radiesse Dilution and Mixing Strategy

Dilutions of Radiesse with activated PRGF (PRGF A) or PRGF Supernatant (PRGF S) were prepared at ratios of 1:1, 1:2, 1:3, 1:4, and 1:6, in accordance with commonly used dilution and hyperdilution patterns encompassing a broad spectrum of clinical applications [10, 17, 19, 36]. The mixtures were prepared at the desired ratios using sterile 3 mL polypropylene syringes connected via Luer‐lock connectors and subsequently homogenized manually for 30 s. The samples diluted in activated PRGF were incubated for 1 h at 37°C to allow PRGF coagulation prior to the In vitro assays.

### 2.3 Ultrastructure Analysis

Scanning electron microscopy (SEM) analysis was conducted for undiluted Radiesse and Radiesse combined with PRGF A, since fibrin polymerization generates a structural network potentially affecting scaffold morphology. Considering that PRGF S does not induce a stable fibrillar architecture and that the soluble fraction is not retained during sample preparation, PRGF S‐based dilutions were not analyzed by SEM. Radiesse, PRGF A and Radiesse: PRGF A samples were placed into 48‐well culture plates and incubated for 1 h to allow complete clot formation. After washing with PBS, membranes were fixed with 2.5% glutaraldehyde (v/v) in 0.1 M sodium cacodylate buffer (pH 7.4). After that, samples were post‐fixed with 1% OsO_4_ (m/v) in 0.1 M sodium cacodylate buffer (pH 7.4), and subsequently washed again. Finally, the samples were dehydrated in ascending ethanol concentrations, critical point dried (Autosamdri 814. Tousimis, Rockville) and gold‐sputtered. Surfaces of samples were examined using a scanning electron microscope (S‐4800; Hitachi, Japan).

### 2.4 Injectability (Extrudability, %) Assessment

Injectability (extrudability, %) was evaluated for undiluted Radiesse and for the Radiesse formulations diluted in PRGF using 3 mL syringes equipped with a 30 G needle (*n* = 3). Injectability was quantified as the percentage of material extruded from the syringe relative to the initial loaded mass based on the methodology suggested by O'Neill with minor modifications [37].

### 2.5 Cell Culture

Commercially sourced primary human dermal fibroblasts (HDFs) (Innoprot, Derio, Spain; P10858, lot: 22110) were cultured in Fibroblast Medium (FM) supplemented with 2% (v/v) fetal bovine serum (FBS), 1% (v/v) FGS, and 1% (v/v) antibiotic solution. Cultures were maintained at 37°C in a humidified atmosphere with 5% CO_2_. Cell viability was routinely evaluated by trypan blue exclusion (Merck KGaA, Darmstadt, Germany) using a hemocytometer. Experiments were conducted with cells at passages 3–4.

### 2.6 Cell Adhesion

The Radiesse: PRGF dilutions previously manufactured were transferred into 48‐well plates at 200 μL per well to ensure that the well surface was fully covered. Undiluted Radiesse, PRGF A and 10% (v/v) PRGF S were also included as control treatments. For assessing cell proliferation, HDFs were seeded at a density of 20 000 cells/cm^2^ in medium containing 0.2% FBS (200 μL). After 24 h of incubation, the serum‐reduced cell culture medium was removed to discard non‐adherent cells. The wells were then washed twice with phosphate‐buffered saline (PBS) and replenished with fresh serum‐free cell culture medium. Mitocondrial activity was determined using WST‐1 reagent as a indirect indicator of cell adhesion (Roche, Basel, Switzerland) according to the manufacturer's instructions. The samples were incubated for 1 h in the presence of 10% (v/v) WST‐1 under standard culture conditions. Absorbance was measured at 450 nm, with background correction at 650 nm. Results were expressed as a percentage relative to the control condition.

### 2.7 Cell Proliferation

Cell proliferation was assessed using the same metabolic activity assay as that described for cell adhesion [38]. Identical controls to those used in the adhesion assay were employed. Cells were seeded at a density of 10,000 cells/cm^2^ and incubated for 72 h under standard culture conditions as outlined above. Subsequently, the serum‐reduced medium was replaced with fresh medium containing 0.2% FBS, and samples were incubated with the WST‐1 cell proliferation reagent for 1 h under standard culture conditions, as previously described.

### 2.8 Gene Expression Analyses

Cells were seeded at a density of 10 000 cells/cm^2^ in 48‐well plates on the previously described Radiesse: PRGF dilutions and cultured for 24 and 72 h, respectively, prior to RNA extraction. Undiluted Radiesse, PRGF A, 10% (v/v) PRGF S in culture medium containing 0.2% FBS and reduced‐serum culture medium containing 0.2% FBS were also included as control treatments.

#### 2.8.1 RNA Extraction and Quality Control (QC) Assessment

For the analysis of gene expression, total RNA was isolated from cultured HDFs using the NucleoSpin RNA Plus purification kit (Macherey‐Nagel, Düren, Germany), according to the manufacturer's instructions. RNA concentration and purity were evaluated using a Biophotometer Plus spectrophotometer (Eppendorf, Hamburg, Germany).

#### 2.8.2 RT‐qPCR Assays

Quantitative real‐time polymerase chain reaction (RT‐qPCR) assays were designed, executed, and reported in accordance with the MIQE (Minimum Information for Publication of Quantitative Real‐Time PCR Experiments) guidelines to ensure reliability, transparency, and reproducibility of the results [39]. A total of 6 genes related to extracellular matrix remodeling and tissue repair processes were included in the present study: *FGF2* (ID: 2247), *CTGF* (ID: 1490), *MMP1* (ID: 4312), *TIMP1* (ID: 7076), *COL1A1* (ID: 1277) and *COL3A1* (ID: 1281). One‐step real‐time quantitative polymerase chain reaction (RT‐qPCR) was performed using the Reliance Multiplex RT‐qPCR Supermix combined with the following Prime PCR probe assays (BioRad, Hercules, CA, USA): *FGF2* (qHsaCEP0058098), *CTGF* (qHsaCEP0024255), *MMP1* (qHsaCEP0055366), TIMP1 (qHsaCEP0050181), *COL1A1* (qHsaCEP0050510), and *COL3A1* (qHsaCEP0053821). As suggested by Brugè et al. [40], GAPDH (ID: 2597) and SDHA (ID: 6389) were selected as stable reference genes for normoxia‐cultured HDFs considering geNorm and NormFinder algorithms (Prime PCR probe assays qHsaCEP0041396 and qHsaCEP0041575, respectively). Assays were conducted in a CFX96 Touch Real‐Time PCR Detection System (BioRad, Hercules, CA, USA), and the thermal cycling conditions were 50°C (10 min) for reverse transcription, 95°C (10 min) for DNA polymerase activation and template denaturation, and 40 cycles at 95°C (10 s) and 60°C (30 s) in a 10 μL reaction volumen as suggested by the manufacturer. For each biological replicate (*n* = 3), a total of three technical replicates were included. Multiplexing was validated experimentally after comparing the Cq values and amplification efficiencies of multiplex and singleplex reactions as recommended by Broeders et al. [41]. RT‐qPCR efficiency was calculated based on six 10‐fold serial dilution assays via the standard curve method [42]. Gene expression analyses were conducted using a computational method based on Anitua et al. [43]. Relative gene expression normalized via the geometric averaging of multiple internal control genes was estimated according to the approaches described by Vandesompele et al. [44] and Hellemans et al. [45]. Control culture conditions (HDFs cultured on plastic surface with FM supplemented with 0.2% FBS) were used as calibrator for calculating ∆Ct values. Results were reported as log‐transformed relative gene expression values normalized to the control group, in line with common practices for presenting gene expression data. This approach facilitates interpretation by expressing changes in gene expression relative to a reference condition (i.e., untreated control or calibrator), regardless of the quantification method employed [44, 46, 47].

### 2.9 Data Analysis

Data processing and statistical analyses were conducted in R computing environment [48] and visualized using the *ggplot2* package [49]. For each response variable, differences between groups were assessed using one‐way analysis of variance (ANOVA). Model assumptions, including normality of residuals and homoscedasticity, were assessed using both graphical diagnostics and formal statistical tests. When these assumptions were not satisfied, a non‐parametric alternative was applied [50]. Post hoc pairwise comparisons versus the reference condition (undiluted Radiesse) were performed using estimated marginal means, with adjustment for multiple testing using the false discovery rate (FDR) procedure [51]. Statistical significance levels were derived from adjusted *p*‐values and categorized as follows: *p* ≤ 0.05 (*), *p* ≤ 0.01 (**), and *p* ≤ 0.001 (***).

## 3 Results

The ultrastructural analysis of undiluted Radiesse and Radiesse diluted with PRGF A revealed significant differences regarding the uniformity of the examined matrices (Figure 1). Fibrin network formation around CaHA microspheres results in their immobilization within a structured matrix, contrasting with the less organized architecture observed in undiluted Radiesse. This process results in an increased interparticle spacing that is dependent on the dilution ratio employed. Considering that Radiesse is a particulate suspension, homogenization represents a critical processing step to ensure a uniform and reproducible material response, particularly at high dilution ratios where a significant reduction in viscosity is observed. In this context, PRGF‐derived clots, membranes, or gel formulations may act as structuring agents, thus enhancing dispersion stability and improving overall matrix homogeneity.

**FIGURE 1:**
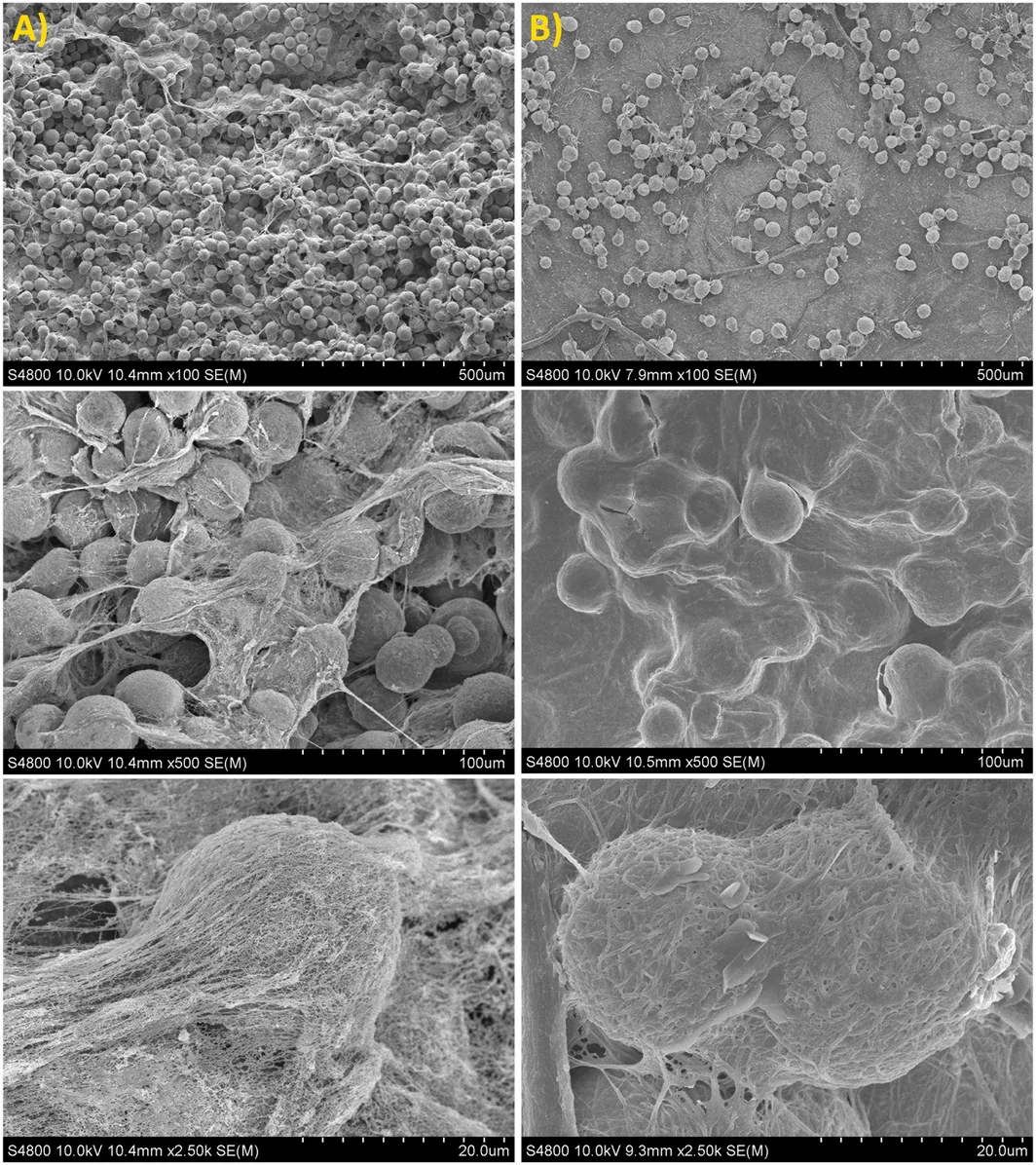
Ultrastructural analysis of undiluted Radiesse and Radiesse diluted with PRGF A. Representative microphotographs at increasing magnifications of the surface of (A) undiluted Radiesse and (B) Radiesse–PRGF A dilutions.

As reported in Table 1, injectability (extrudability, %) remained high across all experimental conditions, ranging from 92.4% to 95.8%. Overall, Radiesse–PRGF mixtures showed injectability values comparable to or slightly higher than undiluted Radiesse, with a modest tendency toward increased injectability at intermediate dilution ratios. Both PRGF A and PRGF S alone exhibited the highest injectability values, suggesting a favorable extrusion behavior of the platelet‐derived fractions. Importantly, no relevant loss of injectability was observed upon dilution of Radiesse with PRGF, even at the highest dilution ratios tested.

**TABLE 1:** Injectability (extrudability, %) of undiluted Radiesse, PRGF formulations, and Radiesse–PRGF mixtures at different dilution ratios.

Group	Injectability (extrudability %)
One Radiesse@: 1 PRGF S	93.1
One Radiesse@: 2 PRGF S	93.8
One Radiesse@: 3 PRGF S	94.2
One Radiesse@: 4 PRGF S	94.9
One Radiesse@: 6 PRGF S	95.3*
One Radiesse@: 1 PRGF A	92.4
One Radiesse@: 2 PRGF A	93.7
One Radiesse@: 3 PRGF A	93.5
One Radiesse@: 4 PRGF A	94.0
One Radiesse@: 6 PRGF A	94.9*
Undiluted Radiesse@	92.9
PRGF A	95.0*
PRGF S	95.8**

*Note:* Significance levels are denoted as *p ≤ 0.05, **p ≤ 0.01, and ***p ≤ 0.001.

Radiesse combined with PRGF significantly increased cell adhesion (28‐fold) compared with undiluted Radiesse (*p* ≤ 0.05) (Figure 2). The highest adhesion values were observed in the 1:2 and 1:6 Radiesse hyperdilutions with PRGF A. This effect may be related to the fibrin network formed upon activation of PRGF, which could provide a more favorable microenvironment for cell adhesion by acting both as a structural scaffold and as a reservoir for bioactive molecules. However, none of the evaluated groups reached the adhesion rate observed in PRGF S and PRGF A.

**FIGURE 2:**
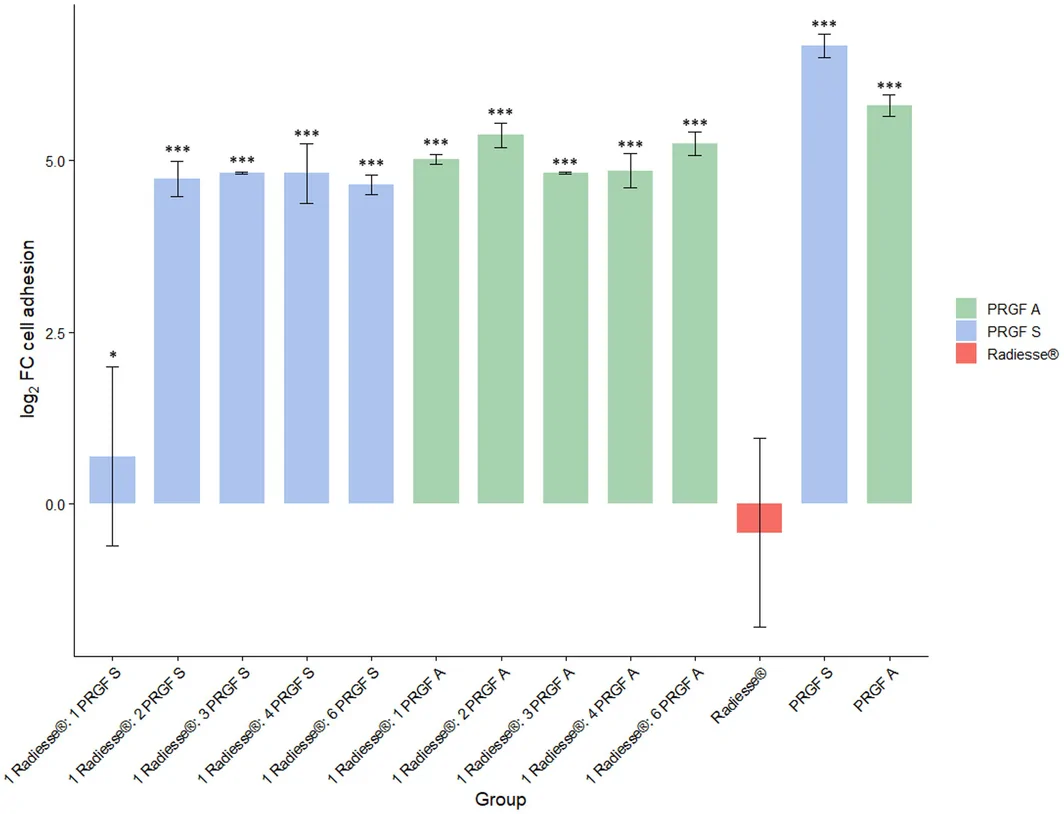
Cell adhesion following exposure to undiluted Radiesse, PRGF and Radiesse:PRGF mixtures at different dilution ratios for 24 h. Radiesse was combined with activated PRGF (PRGF A) or PRGF supernatant (PRGF S) at dilutions ranging from 1:1 to 1:6. Undiluted Radiesse, PRGF A and PRGF S were included as controls. Data are presented as mean log_2_ fold change (log_2_ FC) ± SD. Undiluted Radiesse was used as the reference control for the calculation of fold change values. Significance levels are denoted as *p ≤ 0.05, **p ≤ 0.01, and ***p ≤ 0.001.

As depicted in Figure 3, a significant enhancement in proliferation was observed in cells cultured with Radiesse: PRGF combinations at dilution ratios ranging from 1:1 to 1:6, with increases exceeding 30‐fold compared with undiluted Radiesse (*p* ≤ 0.001). As observed for cell adhesion, PRGF A and PRGF S yielded the highest levels of cell proliferation (*p* ≤ 0.05). An apparent dose‐dependent trend was observed, characterized by a stronger relative effect at lower dilutions and a tendency toward saturation at higher dilutions, despite maximal absolute responses being detected at the highest concentrations tested.

**FIGURE 3:**
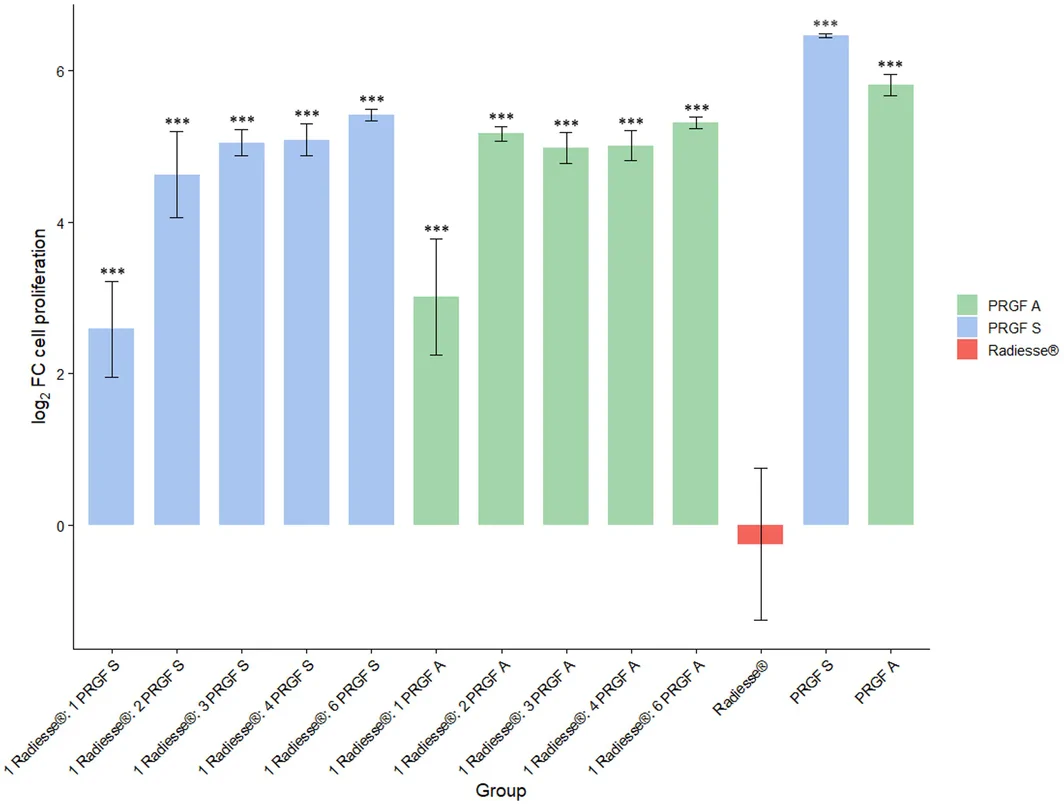
Cell proliferation following exposure to undiluted Radiesse, PRGF and Radiesse: PRGF mixtures at different dilution ratios for 72 h. Radiesse was combined with activated PRGF (PRGF A) or PRGF Supernatant (PRGF S) at dilutions ranging from 1:1 to 1:6. Undiluted Radiesse, PRGF A and PRGF S were included as controls. Data are presented as mean log_2_ fold change (log_2_ FC) ± SD. Undiluted Radiesse was used as the reference control for the calculation of fold change values. Significance levels are denoted as *p ≤ 0.05, **p ≤ 0.01, and ***p ≤ 0.001.

Treatment with undiluted Radiesse elicited a robust transcriptional activation of extracellular matrix–associated genes when compared with the calibrator (Figure 4), as reflected by the significant upregulation of COL3A1 at 24 h (log_2_FC_24h_ = 0.96 ± 0.29; *p* ≤ 0.05) and COL1A1 at 72 h (log_2_FC_72h_ = 1.79 ± 0.46; *p* ≤ 0.05). In parallel, MMP1 was upregulated at 24 h (log_2_FC_24h_ = 1.81 ± 0.28; *p* ≤ 0.01), with a progressive increase in expression over time up to 72 h (log_2_FC_72h_ = 4.98 ± 0.76; *p* ≤ 0.01), whereas TIMP1 initially decreased (log_2_FC_24h_ = −0.83 ± 0.083; *p* ≤ 0.05) and subsequently increased at 72 h, returning to levels similar to the calibrator. Regarding FGF2, transcript levels at 24 and 72 h were comparable to those observed in the control group. Dilution and hyperdilution of Radiesse with PRGF resulted in a significant reconfiguration of gene expression patterns relative to the undiluted condition. These effects were strongly dependent on the Radiesse: PRGF ratio, the incubation time, and the specific PRGF formulation employed, with the supernatant fraction consistently inducing the most pronounced transcriptional modulation. FGF2 was upregulated in the presence of PRGF, particularly after 72 h, with expression levels exceeding those of undiluted Radiesse across the dilution range (*p* ≤ 0.05). Maximum expression levels were observed in PRGF S (log_2_FC_72h_ = 5.55 ± 0.80; *p* ≤ 0.01), whereas PRGF A showed levels comparable to those observed in other PRGF A‐based dilutions (log_2_FC_72h_ = 4.27 ± 0.80; *p* ≤ 0.01). At 24 h, increasing proportions of PRGF were associated with a relative attenuation of FGF2 expression compared with the 1:1 condition (log_2_FC_24h_ = 4.06 ± 0.78; *p* ≤ 0.05), whereas at 72 h the inverse trend was observed, with higher PRGF content corresponding to increased expression levels. Exposure to PRGF S also induced an early increase in MMP1 expression at 24 h compared with undiluted Radiesse (*p* ≤ 0.01), with sustained expression at 72 h reaching levels comparable to those induced by undiluted Radiesse. In contrast, TIMP1 displayed a biphasic expression pattern, with a slight decrease at 24 h in most PRGF‐based dilutions relative to the calibrator (*p* ≤ 0.05) followed by a progressive increase, reaching baseline or above‐baseline levels at 72 h. On the other hand, upregulation of COL1A1 and COL3A1 was restricted to low dilution conditions (Radiesse: PRGF Supernatant 1:1 or Radiesse: activated PRGF 1:1) (*p* ≤ 0.05), followed by a significant decrease at 72 h, indicating a temporally constrained response. In comparison with undiluted Radiesse, all conditions exhibited reduced collagen expression, with the exception of the 1:1 and 1:2 dilutions at 24 h. Additionally, CTGF expression was consistently downregulated in the presence of PRGF A at 72 h when compared with undiluted Radiesse (*p* ≤ 0.05).

**FIGURE 4:**
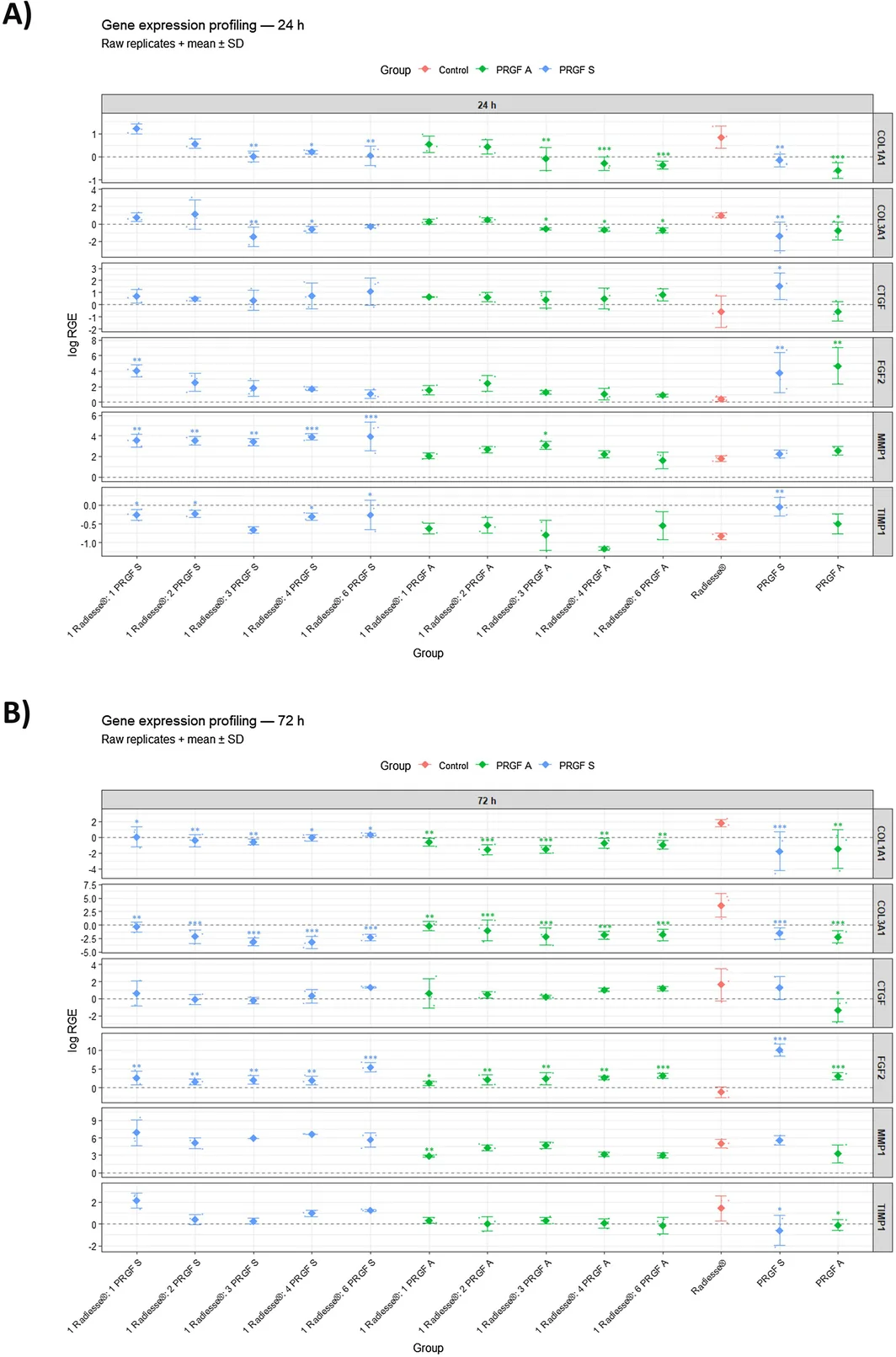
Radiesse: PRGF dilution‐ and time‐dependent transcriptional regulation of key genes involved in ECM remodeling, tissue repair, and regenerative and signaling processes (FGF2, COL3A1, CTGF, MMP1, COL1A1, TIMP1) in HDFs, expressed as log_2_ relative gene expression (logRGE) at 24 and 72 h using reduced‐serum culture medium containing 0.2% FBS as calibrator across different Radiesse: PRGF ratios (1:1 to 1:6), representing commonly used Radiesse dilutions and hyperdilution patterns covering a wide range of clinical applications, including undiluted Radiesse, PRGF A, 10% (v/v) PRGF S in culture medium containing 0.2% FBS and reduced‐serum culture medium containing 0.2% FBS were also included as control treatments. Significance levels corresponding to comparisons between PRGF‐based formulations and undiluted Radiesse are denoted as *p ≤ 0.05, **p ≤ 0.01, and ***p ≤ 0.001.

## 4 Discussion

In the context of regenerative medicine, the integration of biomaterial scaffolds with autologous platelet‐derived biologics represents a promising strategy to enhance tissue repair through complementary biological and structural mechanisms. The significant enhancement in cell adhesion and proliferation observed upon combining Radiesse and PRGF on cell adhesion and proliferation, particularly at hyperdilution ratios (1:2–1:6), is consistent with previous evidence describing the biological activity of PRGF formulations. Upon activation, PRGF has been shown to generate a fibrin network with a highly organized three‐dimensional structure that not only serves as a provisional extracellular matrix that supports complex cell‐matrix interactions but also enables the sustained and localized release of key bioactive mediators, including platelet‐derived growth factor (PDGF), transforming growth factor beta (TGF‐*β*), endothelial growth factor (EGF) or vascular endothelial growth factor (VEGF) [52]. These molecules are well recognized for their central role in regulating fibroblast recruitment, proliferation, and extracellular matrix synthesis, thereby contributing to tissue repair and regeneration processes [53].

Notably, the fact that PRGF alone produced the highest levels of adhesion and proliferation reinforces its central biological role, suggesting that its intrinsic regenerative potential outweighs any contribution from the Radiesse scaffold in early cellular events. Although Radiesse may induce mechanotransduction‐mediated and transcriptional responses of comparable or even greater magnitude, the substantially stronger effects of PRGF on cell adhesion and proliferation irrespective of the specific formulation used as a diluent suggest that early functional outcomes are predominantly driven by its potent bioactive signaling environment, potentially masking scaffold‐mediated effects at the functional level [54, 55, 56, 57]. The substantial increases in cell adhesion and proliferation observed under PRGF‐based conditions can be interpreted as the result of a synergistic combination of a fibrin‐rich scaffold and a bioactive supernatant enriched in platelet‐derived bioactive molecules. From a biological perspective, fibrin provides a native extracellular matrix‐like structure containing integrin‐binding motifs that promote rapid cell attachment, focal adhesion assembly, and cytoskeletal organization, thus facilitating efficient cell spreading and substrate colonization [54]. In parallel, PRGF Supernatant supplies a concentrated pool of soluble mediators, which are well documented to enhance fibroblast recruitment, survival, and proliferative activity [24, 58]. Importantly, literature on platelet‐derivatives consistently supports their role in enhancing both early adhesion and subsequent proliferation by coupling matrix‐dependent mechanotransduction with growth factor–driven mitogenic signaling [59].

These observations were further reinforced by previous reports indicating that PRGF formulations, compared with other platelet concentrates, exhibit a controlled and consistent release of growth factors and other soluble mediators with minimal pro‐inflammatory cytokine content, favoring a pro‐regenerative microenvironment [22, 23]. Overall, these findings suggest that PRGF acts as a major bioactive component at the functional level, contributing to the observed enhancement of cell adhesion and proliferation, and functioning as an autologous biostimulatory system in which soluble mediators and the fibrin scaffold synergistically modulate early cellular responses [60]. Moreover, this strong bio‐stimulatory potential was evidenced by gene expression results, which showed significant modulation of key molecular pathways involved in cellular proliferation and tissue remodeling. In this context, the observed ~30‐fold increase in adhesion and proliferation probably reflects a non‐linear amplification of these converging biomechanical and biochemical cues, rather than a direct transcriptional effect alone. Accordingly, PRGF‐based systems should be understood as highly bioactive microenvironments in which scaffold properties and soluble mediators act in concert to promote robust pro‐adhesive and pro‐proliferative cellular responses. Nevertheless, this large relative difference should be interpreted in the context of the highly concentrated CaHA matrix in undiluted Radiesse, which may limit cell adhesion and contribute to the magnitude of the observed fold‐change. The observed response may also be influenced by factors beyond the intrinsic bioactivity of PRGF, including the physicochemical and rheological changes induced by its incorporation into the CaHA matrix, which may in turn affect cellular accessibility and interaction with the material.

At the transcriptional level, and relative to cells cultured in basal medium, undiluted Radiesse induced a significant upregulation of transcripts associated with extracellular matrix formation, as demonstrated by the upregulation of collagen expression. This phenomenon has been extensively described in the literature, where calcium hydroxylapatite (CaHA)‐based fillers have been shown to induce a sustained biostimulatory effect on the dermal extracellular matrix. In vitro studies have demonstrated that calcium‐based biomaterials, including calcium hydroxylapatite (CaHA)‐based microspheres and related hydroxyapatite formulations, can modulate dermal fibroblast activity through direct cell–material interactions [8, 61, 62]. In particular, exposure of human dermal fibroblasts to CaHA microspheres has been associated with a significant increase in extracellular matrix production, including upregulated synthesis of Type I and Type III collagen, in a process that appears to be both contact‐dependent and time‐dependent, consistent with progressive fibroblast activation and matrix remodeling [16]. These findings were further validated using animal models [63]. Histological studies in human skin have also demonstrated that Radiesse injections stimulate fibroblast activity and promote progressive neocollagenesis [5]. This effect has been clinically observed in association with dermal thickening and long‐term structural matrix remodeling surrounding CaHA microspheres [64]. Collectively, these findings support a mechanical–biological mechanism of action in which CaHA acts as a scaffold that triggers fibroblast activation and extracellular matrix synthesis [1, 65, 66].

At 72 h, collagen Type I expression showed a significant increase, whereas collagen Type III, typically associated with earlier phases of extracellular matrix deposition, reverts to control levels, indicating a transient early response. This occurred concomitantly with a substantial upregulation of MMP1. This pattern may reflect the dynamics of extracellular matrix remodeling, where initial upregulation driven by pro‐fibrotic signals such as TGF‐*β* is followed by compensatory feedback mechanisms, including attenuation of signaling activity and increased MMP‐mediated matrix turnover [67, 68]. Notably, stimulation with undiluted Radiesse did not induce a significant upregulation of CTGF expression. These findings suggest that, despite the observed increase in collagen expression, the transcriptional response may reflect controlled extracellular matrix remodeling rather than sustained profibrotic activation [69]. CTGF is a key downstream mediator of transforming growth factor‐*β* (TGF‐*β*) signaling and plays a central role in fibroblast activation, promoting increased synthesis of extracellular matrix components, particularly collagen Type I, and contributing to sustained tissue repair and remodeling responses [70]. In vitro and tissue engineering studies have demonstrated that CTGF upregulation amplifies fibroblast synthetic activity and stabilizes a profibrotic phenotype, thereby acting as a critical regulatory node in mechanotransduction‐driven matrix deposition processes [71, 72, 73].

Additionally, Radiesse promoted increased expression levels of MMP1 after 24 h together with an upregulation of TIMP1 at 72 h, which returned to levels comparable to the calibrator following an early significant decrease. MMP1 is a principal fibroblast‐derived interstitial collagenase responsible for the degradation of fibrillar collagen within the extracellular matrix [74, 75], whereas TIMP1 is its endogenous inhibitor that regulates matrix metalloproteinase activity and maintains proteolytic balance within the tissue microenvironment [76, 77]. The concomitant upregulation of both genes suggests a coordinated regulation of extracellular matrix turnover pathways following CaHA stimulation, reflecting a tightly controlled remodeling response characterized by concurrent matrix degradation and inhibition of excessive proteolysis. At 72 h, increased MMP1 expression accompanied by a concomitant upregulation of TIMP1 indicates an active extracellular matrix remodeling process characterized by coordinated induction of both proteolytic and inhibitory pathways within the MMP/TIMP axis, consistent with dynamic regulation of matrix turnover during tissue remodeling [78].

The dilution/hyperdilution of Radiesse with PRGF induced significant changes in the expression profiles of the analyzed genes when compared with undiluted Radiesse, reflecting a substantial reprogramming of fibroblast transcriptional activity associated with cell proliferation, collagen biosynthesis and extracellular matrix remodeling–related pathways. Importantly, both the magnitude and the direction (up‐ or downregulation) of these transcriptional responses were strongly dependent on the Radiesse: PRGF ratio and the time point, as well as on the specific composition of the PRGF formulation used as diluent, with PRGF S exerting quantitatively greater changes in overall gene expression, probably due to a higher local availability of bioactive mediators. In particular, the upregulation of FGF2 in response to PRGF at 72 h regardless of the formulation used supports a coordinated shift toward a remodeling phase characterized by coupled extracellular matrix turnover and activation of proliferative and pro‐angiogenic signaling pathways [79, 80]. FGF2 plays a key role in dermal fibroblast activation, tissue repair, and extracellular matrix remodeling [81]. Increased FGF2 expression has been associated with enhanced fibroblast proliferation, migration, and survival, as well as with the regulation of collagen deposition and matrix reorganization during wound healing [58]. In dermal models, FGF2 acts through FGFR‐mediated signaling pathways, including MAPK/ERK and PI3K/AKT cascades, promoting a pro‐regenerative phenotype characterized by controlled extracellular matrix turnover and angiogenic stimulation [82]. Importantly, elevated FGF2 levels have been related to a balanced reparative response rather than fibrotic overactivation, depending on the tissue microenvironment and temporal context of expression [83].

At 24 h, only the 1 Radiesse:1 PRGF S condition resulted in FGF2 expression levels exceeding those of undiluted Radiesse, whereas all other dilution ratios (1:2–1:6) remained comparable to undiluted Radiesse. In contrast, at 72 h this pattern was reversed, with higher PRGF proportions associated with increased FGF2 expression, suggesting a time‐dependent bidirectional regulation of FGF2 signaling consistent with dynamic feedback mechanisms during extracellular matrix remodeling [84]. Simultaneously, as opposed to the later response observed in undiluted Radiesse, PRGF S further stimulated the expression of MMP1 from an earlier time point, indicating enhanced extracellular matrix turnover through controlled collagen degradation and active remodeling dynamics at an earlier phase [85, 86, 87]. Reflecting the transcriptional patterns observed for MMP1, TIMP1 expression was significantly increased at 24 h relative to undiluted Radiesse in most PRGF S‐based dilutions, whereas by 72 h expression levels converged between both conditions, reaching comparable values. In addition, TIMP1 exhibits a biphasic response to PRGF S, with significant upregulation following dilution (1:1) and a reduction at lower ratios to levels comparable to the control at 72 h. A similar expression pattern was observed for structural extracellular matrix components, with significant upregulation of COL1A1 and COL3A1 expression comparable to that induced by undiluted Radiesse occurring exclusively at 24 h under the lowest dilution ratios followed by a significant downregulation at later stages. These findings indicate that PRGF exerts a time‐ and dose‐dependent regulatory effect on collagen expression, attenuating the strong collagen transcription induced by undiluted CaHA in favor of active matrix turnover.

Collectively, these findings indicate that Radiesse: PRGF dilution induced a coordinated and context‐dependent transcriptional response involving key regulators of proliferation, ECM synthesis and remodeling. In particular, the presence of PRGF in combination with Radiesse was associated with increased FGF2 expression, suggesting activation of proliferative signaling pathways involved in tissue plasticity and dynamic regenerative responses. Concomitantly, PRGF modulated the expression of structural ECM components, including COL1A1 and COL3A, by attenuating their sustained expression in a dose‐dependent manner. This indicates a fine‐tuned regulation of collagen expression rather than a unidirectional increase in matrix‐related transcripts as induced by CaHA microspheres. This occurred in parallel with time and context‐dependent changes in MMP1 and TIMP1 expression, reflecting coordinated regulation of ECM turnover through proteolytic and anti‐proteolytic systems. In addition, CTGF expression was not significantly altered following dilution with PRGF, suggesting inhibition of profibrotic signaling and reduced fibrogenesis. The integrated transcriptional response induced by PRGF was characterized by simultaneous regulation of proliferative and remodeling pathways, consistent with a dynamic and plastic extracellular matrix state rather than a linear fibrogenic response. Most transcriptional effects are quantitatively enhanced when PRGF S was used as diluent, potentially reflecting the absence of a fibrin matrix that modulates the release kinetics of signaling mediators. Nevertheless, the interpretation of transcriptional changes, with differences between undiluted Radiesse and its PRGF‐based dilutions ranging from 2‐ to 8‐fold changes in most cases, should be integrated with the substantially amplified functional responses observed under PRGF‐based conditions, where cell adhesion and proliferation increased by up to ~30‐fold.

This indicates that gene expression results alone may not fully capture the overall biological activity, as functional outcomes reflect the integrated effects of signaling networks, post‐transcriptional regulation, and cell–matrix interactions. Consequently, relatively moderate transcriptional changes may translate into disproportionately large functional outputs via non‐linear signaling amplification driven by scaffold‐mediated cell–matrix interactions and local availability of bioactive mediators [54, 88, 89].

## 5 Conclusions

Overall, these experimental results provide a detailed characterization of the rheological properties and In vitro biological effects exerted by different Radiesse dilutions in PRGF, in accordance with dilution ratios commonly used in clinical practice to address a broad range of therapeutic scenarios. The present results suggest that PRGF may be a suitable diluent for Radiesse, offering an autologous component that may contribute to biocompatibility and versatility, enabling the preparation of formulations with different physicochemical compositions, rheological properties, and release kinetics of bioactive molecules. From a biological perspective, PRGF may complement the bioactivity of Radiesse, enhancing cell adhesion and proliferation while inducing transcriptional changes consistent with a pro‐regenerative cellular response, including controlled matrix synthesis and tissue remodeling.

The observed biological effects may reflect the bioactivity of PRGF, changes in the physicochemical and rheological properties of the resulting CaHA formulation, as well as the intrinsic properties of the highly concentrated, undiluted CaHA control used for comparison. However, the relative contribution of these factors remains to be determined. This exploratory study also has limitations inherent to its experimental design, which does not fully reproduce the complexity of In vivo tissue microenvironments. Furthermore, the 24‐ and 72‐h experimental time points were designed to characterize early cellular and transcriptional responses and are insufficient to assess long‐term tissue remodeling or sustained biological effects. Protein‐level deposition of the investigated extracellular matrix markers was not quantified. As a consequence, changes in protein synthesis or deposition cannot be inferred from the RT‐qPCR results, and the molecular findings should be interpreted at the level of gene expression. In addition, the limited number of biological donors may reduce the statistical power and restrict the generalizability of the findings. An additional limitation is the absence of a normal saline‐diluted Radiesse control, which precludes a direct comparison between PRGF and the conventional clinical dilution approach. Further studies including longer experimental time points, larger cohorts, additional clinically relevant control conditions, protein‐level analyses, and In vivo validation are required to further characterize sustained tissue remodeling and corroborate the translational relevance of these findings.

## Author Contributions

Conceptualization: Eduardo Anitua and Mohammad Hamdan Alkhraisat, Methodology: Roberto Tierno and Mohammad Hamdan Alkhraisat, Project administration: Eduardo Anitua and Mohammad Hamdan Alkhraisat, Resources: Eduardo Anitua and Mohammad Hamdan Alkhraisat, Software: Roberto Tierno, Investigation: Roberto Tierno and Mohammad Hamdan Alkhraisat, Formal analysis: Roberto Tierno and Mohammad Hamdan Alkhraisat, Data curation: Roberto Tierno and Mohammad Hamdan Alkhraisat; writing – original draft preparation: Roberto Tierno and Mohammad Hamdan Alkhraisat; writing – review and editing: Eduardo Anitua, Roberto Tierno, and Mohammad Hamdan Alkhraisat, Validation: Roberto Tierno, Visualization: Roberto Tierno and Mohammad Hamdan Alkhraisat, Supervision: Eduardo Anitua and Mohammad Hamdan Alkhraisat, and Funding acquisition: Eduardo Anitua and Mohammad Hamdan Alkhraisat.

## Funding

The authors have nothing to report.

## Disclosure

Medical Writing/Editorial Assistance: The authors confirm that the manuscript was refined using AI‐assisted copy‐editing tools (GPT‐5 mini) to enhance readability, correct grammar/spelling, and improve stylistic flow. Such interventions were exclusively editorial and did not involve any generative content creation, data analysis, interpretation, or authorship. All final edits were reviewed, approved, and remain the original work of the authors, who take sole responsibility for the scientific content, methodology, and conclusions.

## Ethics Statement

All procedures performed in this study were in accordance with the ethical standards of the Araba University Hospital Clinical Research Ethical Committee (BTIIMD‐02‐IV‐23‐NF) and with the Helsinki Declaration as amended in 2024.

## Consent

Consent for treatment and open access publication was obtained or waived by all participants in this study. All procedures performed in this study were in accordance with the ethical standards of the Araba University Hospital Clinical Research Ethical Committee (BTIIMD‐02‐IV‐23‐NF) and with the Helsinki Declaration revised in 2024.

## Conflicts of Interest

The authors declare the following competing financial interests: Eduardo Anitua is the Scientific Director of BTI Biotechnology Institute, and Roberto Tierno and Mohammad Hamdan Alkhraisat are scientists at BTI Biotechnology Institute, a dental implant company that investigates in the fields of oral implantology and PRGF‐Endoret technology.

## Data Availability

The data that support the findings of this study are available from the corresponding author upon reasonable request.
